# The effects of capillary dysfunction on oxygen and glucose extraction in diabetic neuropathy

**DOI:** 10.1007/s00125-014-3461-z

**Published:** 2014-12-16

**Authors:** Leif Østergaard, Nanna B. Finnerup, Astrid J. Terkelsen, Rasmus A. Olesen, Kim R. Drasbek, Lone Knudsen, Sune N. Jespersen, Jan Frystyk, Morten Charles, Reimar W. Thomsen, Jens S. Christiansen, Henning Beck-Nielsen, Troels S. Jensen, Henning Andersen

**Affiliations:** 1 Center of Functionally Integrative Neuroscience and MINDLab, Institute of Clinical Medicine, Aarhus University Hospital, Building 10G, Nørrebrogade 44, DK-8000 Aarhus C, Denmark; 2 Department of Neuroradiology, Aarhus University Hospital, Aarhus, Denmark; 3 Danish Pain Research Center, Institute of Clinical Medicine, Aarhus University, Aarhus, Denmark; 4 Department of Neurology, Aarhus University Hospital, Aarhus, Denmark; 5 Spinal Cord Injury Centre, Department of Neurology, Viborg Regional Hospital, Viborg, Denmark; 6 Department of Physics and Astronomy, Aarhus University, Aarhus, Denmark; 7 Department of Endocrinology and Diabetes, Aarhus University Hospital, Aarhus, Denmark; 8 Department of Clinical Epidemiology, Aarhus University Hospital, Aarhus, Denmark; 9 Department of Public Health, Aarhus University, Aarhus, Denmark; 10 Department of Endocrinology, Odense University Hospital, Odense, Denmark; 11 Institute of Clinical Research, University of Southern Denmark, Odense, Denmark

**Keywords:** Capillary dysfunction, Diabetic complications, Diabetic neuropathy, Glucose intolerance, Glucose transport, Microvascular disease

## Abstract

Diabetic neuropathy is associated with disturbances in endoneurial metabolism and microvascular morphology, but the roles of these factors in the aetiopathogenesis of diabetic neuropathy remain unclear. Changes in endoneurial capillary morphology and vascular reactivity apparently predate the development of diabetic neuropathy in humans, and in manifest neuropathy, reductions in nerve conduction velocity correlate with the level of endoneurial hypoxia. The idea that microvascular changes cause diabetic neuropathy is contradicted, however, by reports of *elevated* endoneurial blood flow in early experimental diabetes, and of unaffected blood flow when early histological signs of neuropathy first develop in humans. We recently showed that disturbances in capillary flow patterns, so-called capillary dysfunction, can reduce the amount of oxygen and glucose that can be extracted by the tissue for a given blood flow. In fact, tissue blood flow must be adjusted to ensure sufficient oxygen extraction as capillary dysfunction becomes more severe, thereby changing the normal relationship between tissue oxygenation and blood flow. This review examines the evidence of capillary dysfunction in diabetic neuropathy, and whether the observed relation between endoneurial blood flow and nerve function is consistent with increasingly disturbed capillary flow patterns. The analysis suggests testable relations between capillary dysfunction, tissue hypoxia, aldose reductase activity, oxidative stress, tissue inflammation and glucose clearance from blood. We discuss the implications of these predictions in relation to the prevention and management of diabetic complications in type 1 and type 2 diabetes, and suggest ways of testing these hypotheses in experimental and clinical settings.

## Introduction

**Fig. 1:**
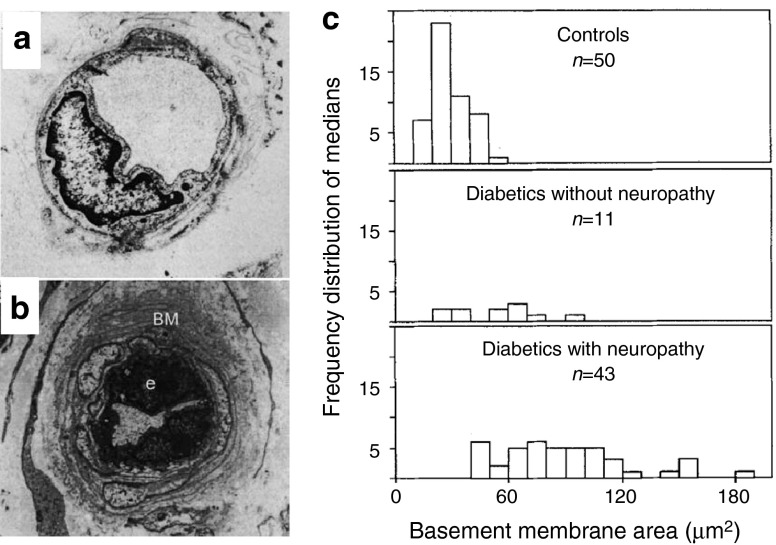
(**a**, **b**) Endoneurial capillaries from the sural nerve of a diabetic patient without neuropathy (**a**) and a patient with neuropathy (**b**). Note basement membrane (BM) thickening and endothelial cell [e] proliferation in (**b**). Reproduced from [6] with permission from the publisher. (**c**) Histograms of the median basement membrane area (in μm^2^) of endoneurial microvessels in 54 diabetic patients (25 with type 1 diabetes, 29 with type 2 diabetes) and 50 controls. The areas were based on transverse electron micrographs of 433 microvessels from diabetic patients and 366 from controls. Note that basement membrane thickening is particularly prevalent in diabetes with neuropathy. Reproduced from Giannini and Dyck [4] with permission from the publisher

Diabetic neuropathy affects up to 50% of patients with diabetes [1]. Distal symmetric sensorimotor polyneuropathy (DNP) is by far the most common form, carrying a high risk of foot ulcers and limb amputation [1]. In addition, one third of patients with neuropathy develop pain, with severe consequences for their quality of life [1, 2]. Despite being the most common complication of diabetes, the pathophysiological mechanisms underlying diabetic neuropathy are largely unknown. The scientific community has generally been divided into two schools of thought, one of which favours a metabolic mechanism, and one proposing a vascular origin of diabetic neuropathy [3]. The latter hypothesis is founded in observations that diabetic neuropathy is associated with microvascular changes in the affected nerve trunks. Nerve biopsies reveal capillary basement membrane thickening, loss of capillary pericyte coverage, and endothelial hyperplasia [4] in endoneurial microvessels (Fig. 1). In fact, changes in endoneurial capillary density and luminal area appear to precede the development of impaired glucose tolerance and diabetes [5]. Nutritive perfusion is reduced in nerve trunks affected by diabetic neuropathy, and their conduction velocities are typically reduced in proportion to the reduction in their oxygen tension [6, 7]. Indeed, changes in vascular reactivity can be recorded prior to the onset of hyperglycaemia in individuals at risk of type 2 diabetes [8]. This ‘vascular’ hypothesis is contradicted, however, by observations that endoneurial blood flow is *elevated* early after the induction of experimental diabetes in rats [9, 10], and observations that sural nerve blood flow in patients with mild diabetes remained constant over a 1-year time period during which nerve fibre density decreased [11]. Meanwhile, several metabolic pathways have been shown to cause nerve damage [12], and it therefore appears that both vascular and metabolic mechanisms may be involved in the pathogenesis of diabetic neuropathy [1, 6].

We recently showed that if capillary flow patterns become disturbed, then the transit times of portions of the blood become too short for oxygen [13] and glucose [14] to be extracted by the tissue. We demonstrated that this ‘physiological shunt’ requires compensatory changes in blood flow to meet the metabolic needs of the tissue and, as a consequence, that tissue may be hypoxic in the absence of demonstrable signs of ischaemia [15]. In this review, we briefly describe the effects of capillary flow disturbances on oxygen and glucose extraction in tissue, and discuss whether capillary changes may contribute to the conflicting endoneurial blood flow findings in early diabetes, to the activity of abnormal metabolic pathways that contribute to diabetic complications, and more generally, to the aetiopathogenesis of glucose intolerance and diabetic complications.

## The relationship between tissue perfusion, capillary transit time heterogeneity and tissue oxygenation

**Fig. 2:**
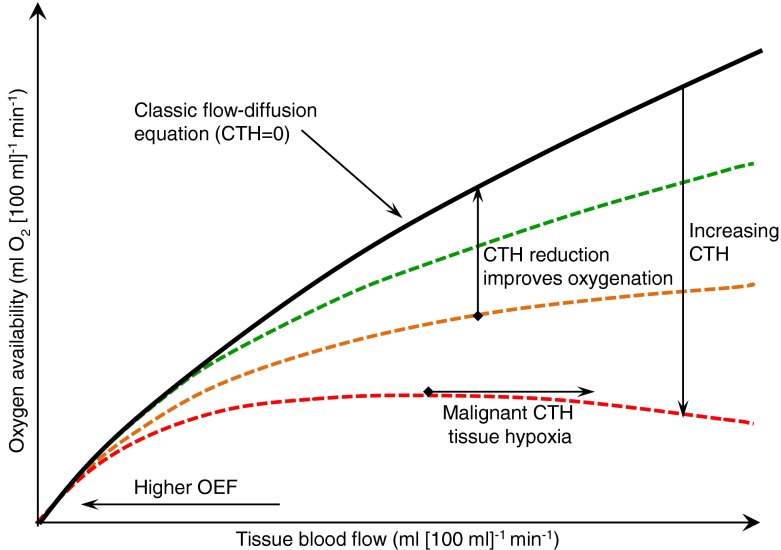
The classic flow-diffusion equation (black curve) describes the relationship between TBF and the amount of a freely diffusible substance, in this case oxygen, that can be extracted by the tissue [13]. The curve is based on the extraction properties of a single capillary with blood flowing through it with a certain velocity. Note that the slope of the curve decreases with flow, indicating that the OEF decreases towards higher TBF. In generalising this relationship to tissue, it was assumed that all capillaries have identical extraction properties. Any deviation from this assumption, in the form of CTH, reduces oxygen availability in relation to the classic flow-diffusion equation’s predictions. In normal tissue, CTH is high during rest but is reduced during hyperaemia. Reductions in CTH improve oxygenation for a given TBF and thereby counteract the tendency for OEF to fall during hyperaemia. If the capillary wall is damaged or blood viscosity increased, CTH may be elevated and fail to homogenise during vasodilation. As a result, TBF increases lead to little improvements in tissue oxygenation, a phenomenon referred to as capillary dysfunction. CTH can become so high that vasodilation no longer improves tissue oxygenation—a combination of TBF and CTH referred to as malignant CTH. From this point, blood flow responses must be attenuated to limit the extent of ‘oxygen shunting’. Continued tissue metabolism tends to lower tissue oxygen tension, thereby increasing blood–tissue concentration gradients and oxygen extraction efficacy. The metabolic needs of nerve function can therefore be supported until the oxygen extraction fraction approaches unity and oxygen tension becomes negligible. Note that, as a result of these biophysical consequences of CTH, both critical reductions in TBF (ischaemia) and critical increases in CTH (capillary dysfunction) can lead to hypoxic tissue injury. Adapted from Østergaard at al [59]

Historically, tissue oxygenation—defined as the maximum metabolic rate of oxygen that can be supported by the blood-stream—is inferred from the flow of oxygenated blood through the tissue. This assumption is rooted in the classic flow-diffusion equation [13], which predicts a one-to-one correspondence between tissue blood flow (TBF; in ml blood per 100 ml tissue per minute) and oxygen availability (ml O_2_ per 100 ml tissue per minute) when arterial blood oxygen content is at normal levels (Fig. 2). This equation assumes, however, that all tissue capillaries are equally perfused. This condition is rarely met in the tissue, where blood velocities normally vary considerably among capillaries—a phenomenon we refer to as capillary transit time heterogeneity (CTH). We recently generalised the flow-diffusion equation to express tissue oxygenation in terms of TBF, CTH, and tissue oxygen tension (P_t_O_2_) [13]. For simplicity, we described the distribution of capillary transit times across the capillary bed by a realistic distribution, for which CTH is simply the standard deviation of capillary transit times, whereas the mean erythrocyte transit time (MTT) is given as the capillary blood volume fraction divided by TBF. Figure 2 shows tissue oxygenation for neural tissue as a function of TBF for different levels of CTH at constant P_t_O_2_. In normal tissue, TBF increases are accompanied by reductions in CTH, which limits ‘physiological shunting’ and maintains efficient oxygen extraction. If capillary function is impaired, however, such that CTH increases and capillary flows fail to homogenise during vasodilation, then increases in TBF lead to little improvement in tissue oxygenation. This phenomenon, dubbed capillary dysfunction, is the result of blood passing through capillaries at transit times too short to permit efficient extraction of its oxygen by the tissue. For capillary dysfunction with modest increases in CTH, the poorer oxygen extraction can be compensated for by higher TBF to meet metabolic needs in tissue, and mild capillary dysfunction is therefore predicted to elicit compensatory tissue hyperaemia. If CTH increases, however, the oxygen loss due to capillary dysfunction can *exceed* the normal oxygenation benefits of vasodilation and hyperaemia. This paradoxical condition is termed ‘malignant CTH’ and is imminent if both CTH and TBF are high: in Fig. 2, this is observed at high TBF levels where oxygenation improves little with further TBF increases because physiological shunting is already high, even for negligible CTH. Therefore, flow increases must be suppressed as TBF approaches the limit of malignant CTH in order to avoid a paradoxical *reduction* in tissue oxygenation. If TBF responses are instead suppressed so that physiological shunting of blood is reduced, then the resulting fall in tissue oxygen tension (the result of ongoing cellular oxygen metabolism) will increase blood–tissue concentration gradients such that more efficient oxygen extraction can further help meet metabolic demands [13].

## The dynamics of blood flow, blood flow responses and tissue oxygen tension as CTH increases

### Mild CTH increase: the hyperaemic state

**Fig. 3:**
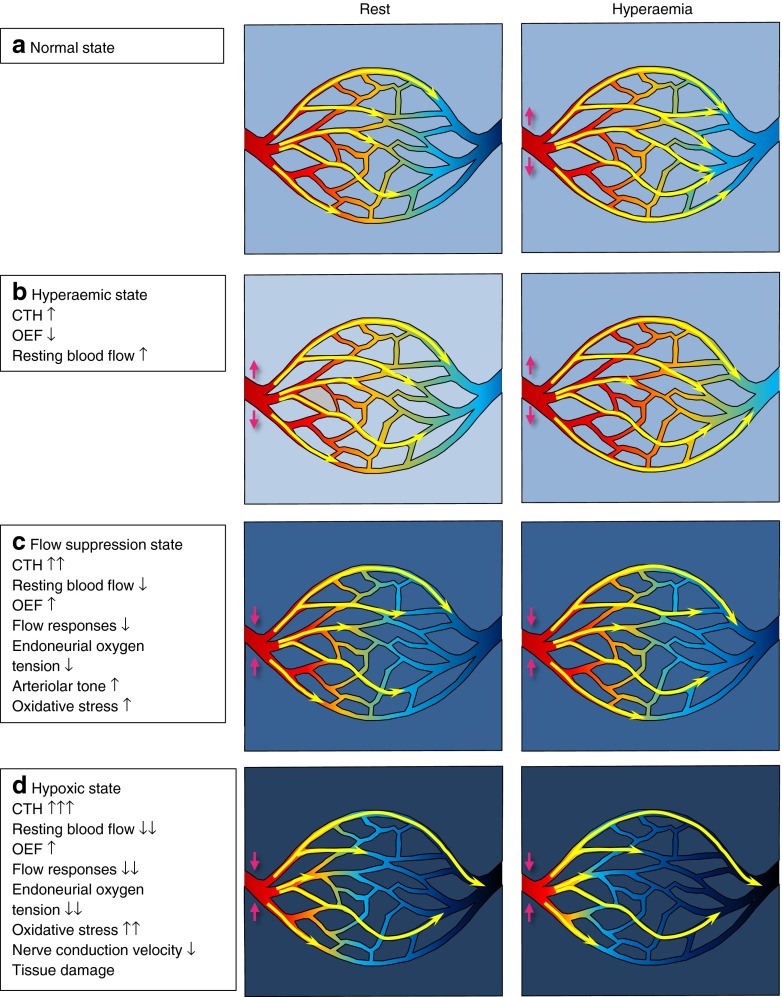
(**a**) Capillary flow patterns homogenise during hyperaemia in normal tissue, counteracting the drop in OEF that would otherwise result from increased tissue blood flow (explained in Fig. 2). If CTH increases and/or fails to homogenise during functional hyperaemia, OEF is reduced. Small reductions in OEF can be compensated by higher flow and/or flow responses to meet the metabolic needs of the tissue. The hyperaemic state (**b**) corresponds to increases in CTH that can still be compensated for by elevated flow and/or flow responses, while the flow suppression state (**c**) corresponds to larger increases in CTH for which resting and/or activity-related flow responses must be suppressed in order to reduce the proportion of blood that passes through the capillary bed too fast to permit efficient oxygen extraction, and to permit the lower tissue oxygen tension (indicated by a darker blue background) to improve blood–tissue concentration gradients, and hence OEF. The suppression of flow responses (endothelial dysfunction) and low tissue oxygen tension is associated with oxidative stress and tissue inflammation. As CTH increases further and oxygen availability and tissue oxygen tension become critically low (dark blue background), the metabolic needs of normal nerve conduction can no longer be met, and nerve function becomes impaired (**d**). The degree of hypoxia in this state is thus predicted to reflect the degree of metabolic impairment and the severity of diabetic neuropathy. Modified from Østergaard et al [59]

Figure 3 summarises the metabolic and haemodynamic consequences of capillary disturbances that elevate flow heterogeneity during rest, and prevent the normal flow homogenisation during hyperaemia. Elevated CTH reduces the maximum oxygen extraction fraction (OEF^max^) that can be attained for a given tissue oxygen tension [13], and the metabolic needs of tissue can therefore be met by slight increases in TBF during rest, and to some extent during activity/hyperaemic challenges. We therefore refer to states of mild CTH increases as hyperaemic.

The findings of increased sciatic blood flow [9, 10] after induction of diabetes by streptozotocin (STZ) in rats are therefore consistent with compensatory increases in blood flow to compensate for poorer oxygen extraction due to subtle changes in capillary flow patterns.

### Moderate CTH increase: the flow suppression state

As changes in capillary morphology or blood rheology accumulate and CTH increases further, increases in TBF can no longer compensate for the parallel reduction in OEF^max^. Instead, TBF must be suppressed to meet the metabolic demands of neural tissue [13]. The blood supply in peripheral nerves originates from two blood supplies: an *extrinsic*, regional vascular system of small arteries and arterioles that connect to epineurial vessels, and a longitudinal, *intrinsic* system characterised by relatively wide endoneurial capillaries [16]. The two systems are interconnected by numerous epineurial and perineurial collaterals that confer considerable resistance to ischaemic damage—see Low et al for a comprehensive overview of peripheral nerve blood flow and metabolism and their relation to nerve damage under diabetic and ischaemic conditions [16]. The tone of epineurial arteries and arterioles is affected by dense perivascular plexuses of noradrenergic, serotonergic and peptidergic nerve fibres, while the intrinsic arterioles display a relative lack of vascular smooth muscle cells [16]. Any reductions in flow or flow responses in relation to increasing capillary dysfunction would therefore be expected to involve epineurial microvessels.

Suppression of endoneurial blood flow and impaired relaxation of epineurial resistance vessels in response to standardised vasodilatory stimuli, so-called endothelial dysfunction, is observed in STZ-induced diabetes in rats prior to any reductions in motor nerve conduction velocity (MNCV) and Na^+^/K^+^ ATPase [17]. Indeed, endoneurial oxygen tension has been observed to decrease *prior to* the decrease in neural blood flow and the onset of neuropathy in STZ-induced diabetes [18], consistent with the prediction that flow suppression represents a compensatory mechanism to ensure sufficient oxygen extraction, rather than the primary cause of nerve dysfunction. Endothelial dysfunction is associated with increased production of reactive oxygen species (ROS) in the vessel wall, and with parallel depletion of the vasodilator NO as it reacts with ROS to produce peroxynitrite [19]. However, oxidative stress [20] and NO depletion [21] are also powerful capillary constrictors. The reversal of endothelial dysfunction and nerve conduction deficits following antioxidant treatment in rats with STZ-induced diabetes [22, 23] may therefore reflect the reversal of *capillary* dysfunction. If capillary dysfunction is irreversible owing to permanent capillary damage, then restoration of nerve blood flow would not be expected to result in improved endoneurial oxygenation. According to this prediction, antioxidant treatment is therefore expected to be less efficacious in disease models and patients with irreversible capillary flow disturbances.

The prediction that increasing CTH is associated with a transition from endoneurial hyperperfusion, when capillary changes are still mild, to normo- and then hypoperfusion when capillary changes become more severe, distinguishes capillary dysfunction from a condition in which blood flow, rather than oxygen extraction, is limited by microvascular changes. Tesfaye et al measured epineurial perfusion in the sural nerve and found reduced blood flow in diabetic patients with chronic sensorimotor neuropathy compared with controls, but increased flow in diabetic patients without neuropathy [24]. These findings are therefore consistent with a progression in capillary dysfunction, with sural nerve *hyperperfusion* (with preserved oxygen supply–demand balance) in diabetic patients prior to the development of neuropathy, progressing to *hypoperfusion* (with oxidative stress and hypoxia) as their neuropathy develops.

### Large CTH increase: the hypoxic state and reduced nerve conduction velocity

If CTH increases even further, the parallel reduction of tissue oxygen tension can contribute to neural tissue dysfunction or damage in several ways. First, the lack of oxygen, and thus of ATP to fuel neural functions, is likely to cause tissue dysfunction. Second, a reduction in tissue oxygen tension upregulates the expression of hypoxia inducible factor 1 (HIF-1) and nuclear factor-κB (NF-κB), both of which are strong pro-inflammatory signals [25]. Indeed, NF-κB levels are elevated in peripheral nerves and dorsal root ganglia in experimental diabetic neuropathy [26], and in humans, both central and peripheral levels of inflammatory markers correlate with the severity of DNP [27]. Third, HIF-1 also upregulates levels of NADPH oxidase 2 (NOX-2) levels [28], a major source of ROS in endothelial dysfunction [29]. ROS in turn react with NO to produce peroxynitrite [19], a source of severe nitrosative tissue damage. In addition, peroxynitrite inactivates tissue plasminogen activator (tPA), consistent with the lack of detectable tPA in endo- and epineurial vessels in patients with diabetic neuropathy [30]. In neuronal tissue, tPA levels determine the formation of brain-derived neurotrophic factor (BDNF) from its precursor, proBDNF. Whereas BDNF is known to provide trophic support for neurons and astrocytes, proBDNF induces neuronal apoptosis [31]. The reduction in distal muscle BDNF and nerve growth factor (NGF) levels indeed correlate with the severity of neuropathy in diabetic patients [32]. This pathway mediates neurodegeneration in diabetic neuropathy [33] and provides a mechanism by which gradual reductions in oxygen availability cause a gradual shift from a state of trophic support for neuronal survival and function to a state of gradual reduction in neuronal fibre number to better match oxygen availability. The relationships between pro-neurotrophins and nociception are discussed in Richner et al [34].

The prediction that nerve fibre function may be supported until the stage where low tissue oxygen tension can no longer secure sufficient oxygen extraction is consistent with the finding that reductions in sural nerve sensory conduction velocity correlate better with nerve oxygen tension than with blood flow values in patients with diabetic neuropathy [35, 36].

**Fig. 4:**
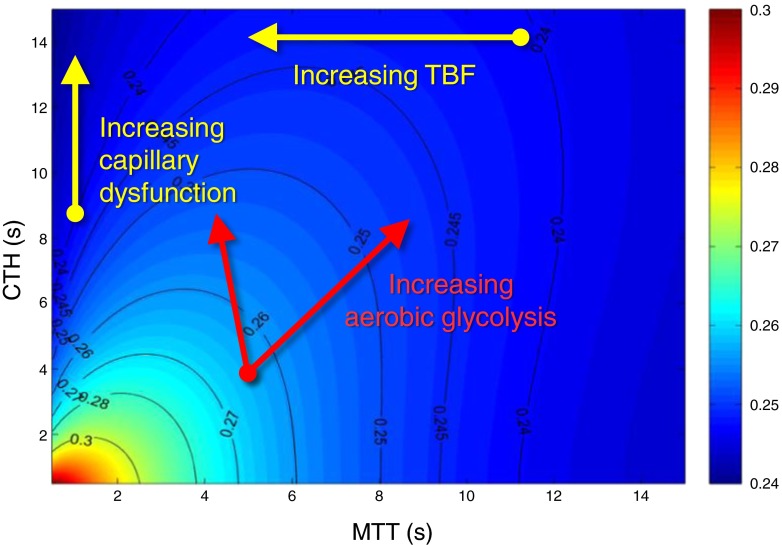
Contour plot showing the relationship between the capillary MTT as blood flows through tissue (*x*-axis), its CTH along the *y*-axis, and the ratio between the net extraction of oxygen and glucose, respectively, as indicated by a colour scale. Warm colours correspond to a high ratio, which permits oxidative phosphorylation to predominate, while blue colours correspond to aerobic glycolysis with limited ATP yields. MTT is defined as the capillary blood volume divided by blood flow, and angiogenesis (which increases capillary density) therefore increases MTT unless blood flow increases in parallel with capillary density. Diabetic angioproliferation tends to produce chaotic microvessels with multiple shunts [37] and would therefore be expected to cause an increase in CTH. The red arrows illustrate two instances of elevated CTH—one in which blood flow increased in parallel with capillary density (left), and one in which upstream microvascular changes prevented TBF changes (right). In both cases, the differential extraction of oxygen and glucose favours lactate formation rather than oxidative phosphorylation. The black lines indicate iso-contours, for which the oxygen:glucose extraction ratio is given by numbers. Adapted from Østergaard et al [14]

Intuitively, one might expect tissue hypoxia to elicit angiogenesis and hence the formation of new capillaries to improve tissue oxygenation. Such capillaries would, however, tend to become immediate shunts for blood that would otherwise pass through capillaries with higher resistance, yet more efficient oxygen extraction [14] (see Fig. 4). Angiogenesis may therefore, paradoxically, exacerbate tissue hypoxia in conditions with pre-existing capillary damage, consistent with reports that insulin neuritis is associated with epineurial microvascular proliferation and excessive arteriovenous shunting [37]. Pericytes are crucial in the initiation of angiogenesis [38]; we speculate that pericyte dysfunction further limits angiogenesis in diabetes.

## ATP production when CTH is elevated: differential effects of capillary dysfunction on oxygen and glucose extraction

Glucose and oxygen are the predominant substrates for the production of the ATP needed for normal peripheral nerve function [39]. The uptake of glucose into the endoneurium is not believed to be insulin dependent [39], but the blood–nerve barrier itself appears to limit the endoneurial extraction of glucose analogues [40]. Unlike rats, humans have few GLUT-1 proteins in endoneurial capillaries [41]. Endoneurial glucose extraction is therefore thought to be limited by the integrity of the blood–nerve barrier rather than by the kinetic properties of glucose transporters [41], and therefore to depend on CTH in much the same way as oxygen extraction. Indeed, indicator dilution studies in the brain show that the extraction of glucose and glucose analogues by the central nervous system is limited by CTH, and that efficient glucose extraction during hyperaemia depends on homogenisation of capillary transit times [42], as discussed for oxygen above. Using these characteristics, the ratio between glucose and oxygen extraction in neural tissue can be assessed, based on the assumption that endoneurial capillaries display a tenfold higher capillary permeability to glucose than those of the brain [40]. This may still be a conservative estimate in that the blood–nerve barrier integrity is disturbed in diabetes, increasing its permeability to glucose and glycosylated serum proteins [43]. Figure 4 shows the ratio between glucose and oxygen extraction as a function of MTT and CTH under these assumptions. Note that, as CTH increases, this ratio is reduced because oxygen uptake is hindered more by CTH and oxygen’s binding to haemoglobin than is glucose. Below, we briefly discuss this aspect of capillary dysfunction in relation to the ATP needs of peripheral nerve function.

Under aerobic conditions, glucose metabolism by oxidative phosphorylation generates 29–30 ATP molecules per molecule of glucose, but when oxygen availability is limited, glucose undergoes anaerobic glycolysis, forming two lactate molecules with an ATP yield of only two—down by a factor of 15 compared with oxidative phosphorylation. In the eye, the kidney, and the myelin sheaths of peripheral nerves, aldose reductase enzymes are present, allowing the conversion of glucose into sorbitol without using the two ATP molecules that are required during the initial phosphorylation step of oxidative phosphorylation. The aldose reductase pathway can therefore preserve endoneurial ATP when oxygen extraction is limited by capillary dysfunction, in that it reduces the ATP expenditure needed to maintain energy-efficient oxidative phosphorylation. However, capillary dysfunction is also predicted to reduce glucose extraction, making less glucose available for the aldose reductase pathway. Thus, ATP production via this pathway may be insufficient to secure normal peripheral nerve function. The possible links between nerve energy status (hypoxia, low pH and increased lactate levels) and pain are discussed in more detail below.

We note that, according to this prediction, increased utilisation of the aldose reductase pathway in diabetes is a result of increasing capillary damage, rather than of high blood glucose per se. In particular, inhibition of fructose formation via this pathway would be expected to *worsen* the energy crisis of peripheral nerves in diabetes by favouring glucose metabolism via the more ATP-demanding phosphorylation pathway. This is consistent with findings that sorbitol dehydrogenase inhibitors fail to increase nerve blood flow or conduction velocity in experimental diabetes [44] but instead worsen nerve energy status in some studies [45], and apparently exacerbate sympathetic autonomic neuropathy in STZ-induced diabetes [46].

Despite its ability to preserve ATP production for nerve function, activation of the aldose reductase pathway is known to have deleterious long-term effects: the conversion of glucose into sorbitol uses NADPH, and the subsequent conversion of sorbitol into fructose uses NAD, both of which alter the cell redox state [47]. In particular, NADPH is important for the regeneration of reduced glutathione, an important reactive oxygen species (ROS) scavenger. Long-term activation of the aldose reductase pathway is therefore expected to cause oxidative damage to peripheral nerves [47]. Aldose reductase pathway inhibition is therefore potentially a double-edged sword in that it reduces oxidative stress on the one hand, while exacerbating tissue energy crisis on the other by causing a shift to less ATP-efficient glucose metabolism. We speculate that these effects may explain why aldose reductase inhibitors have attenuated the progression of neuropathic changes in some clinical trials, while failing to do so in others [48, 49].

### Capillary damage and oxidative stress due to hyperglycaemia

Hyperglycaemia causes the formation of AGEs via non-enzymatic reactions between aldehyde groups of reducing sugars with proteins, lipids and nucleic acids. The production of AGEs is associated with ROS production, just as AGEs interact with the AGE receptor (RAGE) causing further ROS release [29, 47], and consequently oxidative damage.

Hyperglycaemia [50], oxidative stress and oxidised lipoproteins [51, 52] disrupt the glycocalyx, a 0.5 μm thick carbohydrate-rich matrix that covers the luminal surface of the capillary endothelium [53]. The glycocalyx is thought to play a key role in the control of erythrocyte flow through the capillary bed [54], and its disruption is therefore likely to cause capillary dysfunction. Underscoring this regulatory role, glycocalyx disruption causes an increase in capillary haematocrit from only 20–50% of full blood haematocrit, to approach values similar to those found in the systemic circulation [52]. Mice fed a high-fat diet to generate high levels of oxidised lipoprotein and oxidative stress develop reduced nerve conduction velocities and sensory deficits *before* glucose tolerance is impaired [55]. This finding is consistent with a role of generalised capillary dysfunction in the development of glucose intolerance, alongside the development of reduced nerve conduction velocity as a result of endoneurial capillary dysfunction and hypoxia; see below.

## Sources of pain in diabetic neuropathy

While progressive changes in capillary morphology and function may cause reductions in nerve function and even nerve damage, these mechanisms fail to explain the mechanical hyperalgesia and tactile allodynia (sensation of pain in response to otherwise non-painful stimuli) experienced by nearly half of patients with diabetic neuropathy [3]. Endoneurial hypoxia is associated with upregulation of HIF-1 and NF-κB [25] (see above). NF-κB is crucial in the regulation of developmental and synaptic plasticity and can prevent the death of neurons by the production of anti-apoptotic proteins [56]. While the activation of NF-κB may thus serve to protect nerve integrity and function under conditions of hypoxia, it also appears to be involved in neuropathic and inflammatory pain [57]. Notably, sulfasalazine reduces the expression of NF-κB p50 in both sciatic nerves and dorsal root ganglia of STZ diabetic rats, blocking their development of tactile allodynia [58]. The relationships between pro-neurotrophin levels (above) and nociception are discussed in detail elsewhere [34], and the relation between tissue injury, tissue hypoxia and pain, in Østergaard et al [59].

Painful diabetic neuropathy may be related to the function of small, autonomic fibres in diabetic patients. Small fibre dysfunction with sympathetic denervation of the peripheral arterial system is thought to occur quite early in the progression of neuropathy [60]. The resulting loss of vasoconstrictor tone and peripheral vasodilatation gives rise to the appearance of a warm, oedematous neuropathic foot [60]. The high peripheral blood flow passes through arteriovenous shunts [60], and it was recently hypothesised that excessive microvascular shunting may give rise to tissue hypoxia, despite the high blood flow [61]. Our model of oxygen extraction in tissue [13] supports this notion, and predicts that failure to suppress blood flow in capillary dysfunction can be the source of severe oxidative stress, microvascular injury and pain [59]. Archer et al [62] showed that blood flow in the feet of patients with diabetic neuropathy is five times higher than in normal controls. While patients with painful diabetic neuropathy had slightly lower blood flow than those without pain, the groups differed further by the preserved ability of sympathetic stimuli to suppress blood flow in the group with painful diabetic neuropathy. Furthermore, reductions in blood flow were associated with a reduction in neuropathic pain, similar to the pain relief reported by some patients when cooling the feet (which would be expected to cause local vasoconstriction) [62]. Taken together, these observations support the role of hypoxia in painful diabetic neuropathy and suggest that small sympathetic fibres play a role in the pain mechanism, possibly in relation to their vasomotor action under conditions where suppression of peripheral blood flow appears important to meet the metabolic needs of the tissue.

## The role of capillary pericytes in diabetic complications

Loss of pericytes is evident in biopsy material from patients with diabetic neuropathy [4], and pericyte loss is closely related to the severity of diabetic retinopathy [63, 64]. In the central nervous system [65, 66] and the retina [67–69], pericytes regulate capillary diameter according to local metabolic needs. Pericytes and endothelial cells form the capillary basal membrane [38], which (in addition to those of peripheral nerves) is thickened in several organs in diabetes [70, 71]. Based on their proposed role in maintaining efficient oxygen extraction, means of supporting pericyte function and survival might therefore be expected to alleviate diabetic neuropathy. This notion is in agreement with animal models of diabetic retinopathy, where the development of retinal damage appears to be closely related to pericyte apoptosis [64]. Of note, the rescue of retinal pericytes was recently shown to prevent diabetic retinopathy in animal models [64].

The control of pericyte tone remains much less studied than that of arteriolar tone [72]. Studies of retinal capillaries suggest that pericytes react to intrinsic signalling in much the same way as smooth muscle cells. Pericyte constriction has been observed in response to mechanical stretch, exposure to angiotensin II (via AT_1_ receptors) [73], and endothelin-1 (via ET_A_ receptors) [74], by a Ca^2+^ dependent mechanism [75]. Retinal pericytes relax in response to NO [21] and adrenergic (via β_2_ receptors) [75] stimulation. In cerebral pericytes, ischaemia and oxidative stress cause irreversible capillary constriction [20, 66].

Restoration of capillary NO levels would be expected to improve CTH (homogenising capillary flow patterns) by facilitating pericyte relaxation. This may be achieved in ways that do not require oxygen as a substrate for NO synthesis, namely by dietary administration of nitrate or nitrite, which is readily converted to NO in the tissue [76]. Green leafy vegetables are sources of dietary nitrate and seemingly reduce the risk of developing type 2 diabetes [77]. Meanwhile, topical application of nitrate reduces neuropathic pain and burning sensation, but not other sensory modalities, in patients with painful diabetic neuropathy [78]. Pharmacologically, antihypertensive drugs would be expected to modulate the effects of angiotensin and endothelin on pericyte tone, or its Ca^2+^ dependent regulation. ACE inhibitor treatment has been shown to improve nerve conduction, but not autonomic function, vibration perception threshold, or neuropathy symptom and deficit score, in normotensive diabetic patients [79]. Furthermore, ACE inhibitor and angiotensin II receptor antagonist treatment improved nerve conduction velocities, reduced oxidative stress, and reverted endoneurial flow suppression in STZ mice [80]. Interestingly, ACE inhibitor administration prior to the induction of diabetes by STZ in rats was found to prevent development of nerve conduction abnormalities [81]. Calcium blocker treatment has also been reported to reverse flow suppression in the vasa nervorum of STZ diabetic rats [82], and to improve their motor- and sensory nerve conduction velocity [83].

## Potential implications for diabetes management

Our review suggests that early loss of capillary flow control and changes in capillary morphology may play a central role in the aetiopathogenesis of diabetic neuropathy.

The prediction that elevated CTH impairs both oxygen and glucose extraction in tissue also implies that strategies to prevent diabetic complications may differ between patients with type 1 and type 2 diabetes, respectively. The diagnosis of type 1 diabetes marks the onset of hyperglycaemia-related capillary damage to peripheral nerves (as explained above) and organ microvasculature in general, to an extent that would be expected to depend on the cumulative exposure to hyperglycaemia. Early, intensive glycaemic control indeed delays the onset of type 1 diabetes complications, including diabetic neuropathy [84]. By contrast, type 2 diabetes and its associated complications, risk factors such as age, obesity and hypertension, are all associated with either degenerative changes in capillary morphology [85] or dysfunctional angiogenesis [86] prior to the onset of type 2 diabetes. Indeed, given the effects of CTH on glucose clearance from blood, the progressive capillary dysfunction of the systemic microcirculation caused by type 2 diabetes risk factors is likely to reduce glucose tolerance, and hence contribute to what we define as type 2 diabetes. We therefore suggest that type 2 diabetes complications represent the progression of systemic capillary dysfunction from more moderate levels *already present* when type 2 diabetes is diagnosed. This is consistent with recent observations of early small fibre loss in the cornea of patients with impaired glucose tolerance [87] and recently diagnosed type 2 diabetes [88], keeping in mind that while the cornea is avascular, the proximal course of its fibres depend on capillary function to maintain function and trophic support.

The prediction that hyperglycaemia is one of many sources of capillary dysfunction in type 2 diabetes suggests that its comorbidities and risk factors, including hypertension, systemic inflammation, hypercholesterolaemia and smoking, should be viewed and managed as separate, modifiable sources of additional capillary dysfunction. (1) In angiotensin II models of hypertension, flow responses are indeed attenuated in some organs prior to the development of increased blood pressure [89], suggesting that the increased peripheral resistance in hypertension represents a systemic response to preserve tissue oxygenation in response to widespread capillary/pericyte constrictions and elevated CTH in response to this powerful pericyte constrictor. (2) Animal studies of systemic inflammation have shown that capillary flow patterns are sensitive to the size, viscosity, number and endothelial adhesion of blood cells, and undergo profound changes as part of the low-grade vascular inflammation that accompanies many cardiovascular risk factors [51, 90]. In diabetic patients, blood viscosity at low shear rates is indeed elevated, correlating with the extent of their microvascular diabetic complications [91]. See also a discussion of blood viscosity changes in diabetes in Low et al [16]. (3) Plasma lipid levels also affect blood viscosity, and high triacylglycerol and cholesterol levels are therefore predicted to represent an independent risk factor for type 2 diabetes and its complications, while lipid-lowering therapy would be predicted to reduce CTH and hence improve endoneurial oxygenation while reducing oxidative damage and the development and progression of diabetic neuropathy. This is consistent with observations that triacylglycerol levels correlate with the progression of diabetic neuropathy [92], with clinical trials [93], cohort studies [94, 95] showing benefits of statin treatment in type 2 diabetes, and with animal studies showing restoration of vasa nervorum function and reversal of diabetic neuropathy after statin treatment [96]. Importantly, fibrates (which lower triacylglycerol and cholesterol levels) and statins seem more efficient than intensive blood glucose control in reducing the rate of amputation in type 2 diabetes [97]. While plasma viscosity may represent a putative target for diabetes management, we propose that observations of neuropathic pain severity during infections, where leucocytosis causes capillary flow patterns to become more disturbed [90], would serve as an indirect confirmation of the role of capillary dysfunction in diabetic neuropathy. (4) Nicotine upregulates the expression of adhesion molecules in the capillary endothelium [98] and increases leucocyte rolling [99], consistent with findings that smoking represents an independent risk factor for diabetic neuropathy [100]. Cessation of nicotine exposure would therefore be predicted to alleviate both symptoms and progression of diabetic neuropathy. Similarly, high homocysteine levels increase blood viscosity and the adhesion of monocytes to the capillary wall, and increase the oxidation of low-density lipoproteins [101] (see the section on glycocalyx function above). These effects would be expected to cause capillary dysfunction and progression of neuropathy, consistent with reports that homocysteine is independently associated with diabetic neuropathy in patients with type 2 diabetes [102]. Similarly, findings of more severe neuropathy in type 2 diabetic patients who had received metformin may be related to the accompanying increases in blood homocysteine levels, in addition to the effects of long-term reduction in cobalamine (vitamin B_12_) levels [103].

## Conclusion

The proposed hypothesis that capillary dysfunction causes diabetic neuropathy (and some degree of glucose intolerance) gives rise to a range of predictions that lend themselves to further scrutiny in animal experiments, epidemiological studies and clinical trials. The hypothesis relates type 2 diabetes risk factors, and effects of poor glycaemic control on capillary function in both type 1 and type 2 diabetes, to their effects on blood rheology and the morphology and function of capillaries. Accordingly, we predict that animal models of diabetic complications should display capillary dysfunction or damage similar to that observed in human nerves, kidney and retina in order to predict the translational potential of experimental therapies. Pericyte function and pericyte damage also appear to be important to our understanding of diabetic neuropathy.

Capillary dysfunction is summarised in a single parameter, CTH, which we propose determines the derived effects on extraction of oxygen and glucose in various tissue types. So far, studies of diabetic neuropathy have focused on nerve blood flow rather than its capillary distribution. To extend the indirect evidence of capillary dysfunction presented here, capillary flow velocities [104] and nerve oxygen tension [105] must therefore be imaged longitudinally and related to nerve function in animal models of diabetes. To translate such studies into human disease, microvascular flow distributions and CTH may be estimated noninvasively by dynamic tracking of intravascular contrast agent retention after bolus injection [106], using, for example, contrast enhanced ultrasound to capture the haemodynamics in peripheral nerves.

## Abbreviations

BDNF: Brain-derived neurotrophic factor
CTH: Capillary transit time heterogeneity
DNP: Distal symmetric sensorimotor polyneuropathy
HIF-1: Hypoxia inducible factor 1
MTT: Mean erythrocyte transit time
NF-κB: Nuclear factor-κB
OEF: Oxygen extraction fraction
ROS: Reactive oxygen species
STZ: Streptozotocin
TBF: Tissue blood flow
tPA: Tissue plasminogen activator
